# Timeframe Analysis of Novel Synthetic Cannabinoids Effects: A Study on Behavioral Response and Endogenous Cannabinoids Disruption

**DOI:** 10.3390/ijms25063083

**Published:** 2024-03-07

**Authors:** Jorge Carlos Pineda Garcia, Ren-Shi Li, Ruri Kikura-Hanajiri, Yoshitaka Tanaka, Yuji Ishii

**Affiliations:** 1 Laboratory of Molecular Life Sciences, Graduate School of Pharmaceutical Sciences, Kyushu University, 3-1-1 Maidashi, Higashi-ku, Fukuoka 812-8582, Japan; jturilli@hotmail.com (J.C.P.G.); li-renshi@cpu.edu.cn (R.-S.L.); 2 Division of Pharmaceutical Cell Biology, Graduate School of Pharmaceutical Sciences, Kyushu University, 3-1-1 Maidashi, Higashi-ku, Fukuoka 812-8582, Japan; ytanaka@phar.kyushu-u.ac.jp; 3 School of Traditional Chinese Pharmacy, China Pharmaceutical University, Nanjing 210009, China; 4 Division of Medicinal Safety Science, National Institute of Health Sciences, 3-25-26 Tonomachi, Kawasaki-ku, Kawasaki 210-9501, Japan; kikura@nihs.go.jp

**Keywords:** MDMB-CHMINACA, 5F-ADB-PINACA, APICA, JWH-018, locomotive, memory, behavior, indazole/indole–carboxamide

## Abstract

This study investigates the impact of SCs consumption by assessing the effects of three novel synthetic cannabinoids (SCs); MDMB-CHMINACA, 5F-ADB-PINACA, and APICA post-drug treatment. SCs are known for their rapid onset (<1 min) and prolonged duration (≥5 h). Therefore, this research aimed to assess behavioral responses and their correlation with endocannabinoids (ECs) accumulation in the hippocampus, and EC’s metabolic enzymes alteration at different timeframes (1-3-5-h) following drug administration. Different extents of locomotive disruption and sustained anxiety-like symptoms were observed throughout all-encompassing timeframes of drug administration. Notably, MDMB-CHMINACA induced significant memory impairment at 1 and 3 h. Elevated levels of anandamide (AEA) and 2-arachidonoyl glycerol (2-AG) were detected 1 h post-MDMB-CHMINACA and 5F-ADB-PINACA administration. Reduced mRNA expression levels of fatty acid amide hydrolase (FAAH), monoacylglycerol lipase (MAGL) (AEA and 2-AG degrading enzymes, respectively), and brain-derived neurotrophic factor (BDNF) occurred at 1 h, with FAAH levels remaining reduced at 3 h. These findings suggest a connection between increased EC content and decreased BDNF expression following SC exposure. Cognitive disruption, particularly motor coordination decline and progressive loss manifested in a time-dependent manner across all the analyzed SCs. Our study highlights the importance of adopting a temporal framework when assessing the effects of SCs.

## 1. Introduction

The identification of cannabinoid receptors type 1 (CB_1_ receptor) and type 2 (CB_2_ receptor) [1,2] along with their major endogenous ligands, anandamide (AEA) and 2-arachidonoyl glycerol (2-AG), also known as endocannabinoids (ECs) [3,4,5], ignited a strong interest in understanding the implication of ECs alteration in the modulation of physiological processes such as neurodegenerative disorders, inflammation, nausea, appetite, mood, and metabolism, among others [6,7,8]. The discovery of the enzymes involved in EC’s synthesis, release, transport, and degradation helped to further elucidate the interrelated system among these ECs, their metabolic enzymes and both CB receptors [9,10,11,12]. Together, this intricate system is referred to as the endocannabinoid system [13,14]. Consequently, studies focused on identifying possible therapeutic substances to regulate physiological processes via the endocannabinoid system were vigorously pursued, leading to the development of certain substances that exhibited a high affinity towards CB receptors. These substances were categorized as synthetic cannabinoids (SCs) [8,15,16]. Since then, the use of SCs has undergone dramatic changes.

Synthetic cannabinoids (SCs) have been regarded as potential therapeutic agents because of their high cannabimimetic potency and stereoselectivity for the CB_1_ and/or CB_2_ receptors [15,16]; however, the elimination of unwanted psychotropic effects similar to those of Δ^9^-tetrahydrocannabinol (Δ^9^-THC) from their possible therapeutic properties remains difficult to achieve [17].

As a result, the majority of these substances have not been incorporated into medical treatment and their development as candidate drugs in the pharmaceutical industry has been discontinued [17,18]. Despite their rejection as potential therapeutic agents, the methods of synthesis have been published in the scientific literature, enabling clandestine chemists to produce and further commercialize SCs in the cannabis and drug user communities [19]. Two features make SCs their drug of choice: (1) the rapid onset of action and (2) their prolonged psychoactive effects for up to 5 or more hours [20]. Additionally, their composition is never the same as both their dosage and components fluctuate according to the producer. Therefore, the extent of their efficacy, potency, and duration of action varies according to the SC of choice [21]. For this reason, SCs are the most popular and rapidly growing class of designer drugs [22,23] and are currently used mostly as recreational drugs of abuse [24,25], exacerbating public health concerns [22].

**Figure 1:**
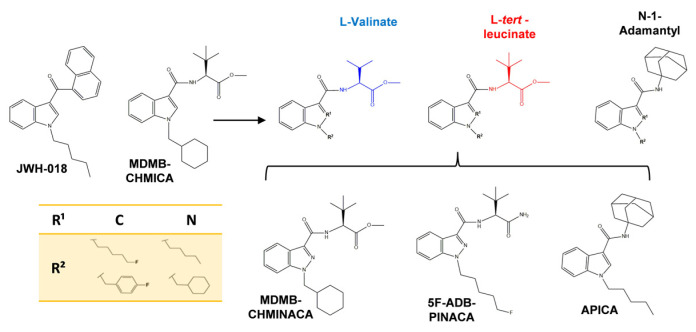
Schematic representation of the chemical structures and substituents of indole/indazole derivatives synthetic cannabinoids; MDMB-CHMINACA, 5F-ADB-PINACA, APICA. The ease of insertion of an additional N atom into the heterocyclic system of the classic synthetic cannabinoid JWH-018 and the archetypal aminoalkylindole-based synthetic cannabinoid MDMB-CHMICA structures along with the presence of a carboxamide group, diverse substituents on both the N-1 atom of the indazole ring, and the carboxamide N atom promote the synthesis of many indazole-3-carboxamide based SCs. They usually comprise an indazole-carboxamides or N-1-adamantyl indole-carboxamide at the 3-position. Adapted from [29].

The current research focused on three novel SCs with indazole/indole-3-carboxamide cores, N-[[1-(cyclohexylmethyl)-1H-indazol-3-yl]carbonyl]-3-methyl-L-valine, methyl ester (MDMB-CHMINACA), N-(1-amino-3,3-dimethyl-1-oxobutan-2-yl)-1-(5-fluoropentyl)-1H-indazole-3-carboxamide (5F-ADB-PINACA), and N-(1-adamantyl)-1-pentyl-1H-indole-3-carboxamide (APICA). They are derived from the aminoalkyl–indoles/indazoles class, the largest and most prevalent group of SCs [26,27]. Their side chain comprises L-valinamide, tert-leucinamide, and adamantyl groups, respectively, which confer them a varied spectrum of pharmacological outcomes, selectivity, and stability patterns [28,29]. They are also structurally related to MDMB-CHMICA, a 10 times more potent SC analog of JWH-018, the first SC which was considered as the basis for further SCs development [26,30] (Figure 1).

SCs bearing an indazole/indole-3–carboxamide core have analogous conformation; however, they have different affinities for CB_1_ and CB_2_ receptors. For instance, MDMB-CHMINACA and 5F-ADB-PINACA have a greater affinity for CB_1_ receptors (Ki: 0.135 nM, Ki: 0.55 nM, respectively), whereas APICA is more selective towards CB_2_ receptors (Ki: 1.22 nM) [31,32]. Furthermore, the measurement of their standard pharmacokinetics and pharmacodynamics parameters is challenging due to their diverse structures, rapid, extensive, and variable metabolism, and unconventional production methods [33,34]. The biotransformation of parent drugs and metabolites occurs within the first 5 h of consumption. Notably, two major active metabolites targeting CB_1_ receptors have been reported for MDMB-CHMINACA, while 5F-ADB-PINACA reports four active metabolites. In contrast, APICA metabolites predominantly interact with the CB_2_ receptors, being hydroxylation, carboxylation, and glucuronidation, the predominant metabolic pathways of these SCs [28,29,33,35,36].

Our laboratory previously reported that JWH-018 impaired memory and learning by elevating the endogenous brain cannabinoids (ECs) 2-AG and AEA in response to the suppression of their degrading enzymes MAGL and FAAH, respectively. In addition, hyperactivity and anxiety have been observed 2 h after drug administration [37]. Moreover, indazole/indole–carboxamide SC derivatives induce abnormal and aggressive behavior [38].

**Figure 2:**
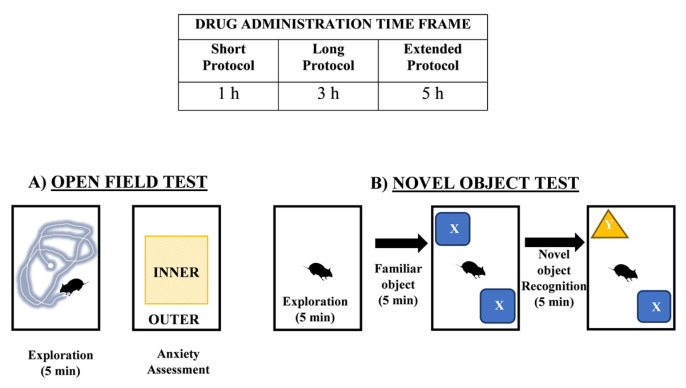
Schematic diagram of behavioral studies. (**A**) Outline of the exploration trial and anxiety assessment configuration. (**B**) Layout of the recognition trial arena tests. Adapted from [39].

While the acute effects of SCs are well-documented, research exploring the extent of their cognitive impairment is still ongoing. We postulate that exposure to structurally similar SCs, each exhibiting distinct affinities for either CB_1_ or CB_2_, may induce varying degrees of behavioral disruption and diverse alterations in hippocampal ECs levels. To investigate this, we exposed test subjects to different timeframes of SC administration—MDMB CHMINACA, 5F-ADB-PINACA, and APICA―and assessed their behavioral, memory and learning responses at short term (1 h), long term (3 h), and extended term (5 h) intervals. This approach allowed us to monitor and evaluate adverse effects and behavioral changes within a timeframe aligned with the intrinsic metabolism and pharmacological dynamics of SCs (Figure 2A,B).

By employing a timeframe-oriented methodology, we aim to offer valuable insights into the dynamic pharmacological effects of SCs and the progression of cognitive impairment linked to alterations in hippocampal EC levels following SC administration. The overall purpose of this study is to determine whether the elevation of ECs represents an inherent and shared trait among SCs and whether it aligned with the severity of cognitive impairment. Consequently, this study sought to categorize an indicative state of SC drug abuse.

## 2. Results

### 2.1. Behavioral Response Studies

#### 2.1.1. Distance Travelled and Velocity

**Figure 3:**
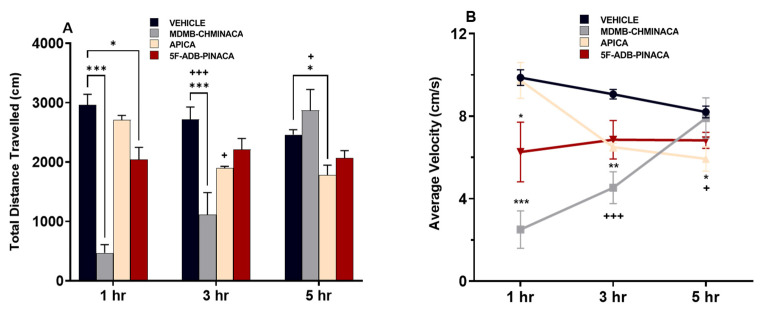
Effects of 1 mg/kg systemic administration of MDMB-CHMINACA, APICA, and 5F-ADB-PINACA on the (**A**) total distance traveled (cm) and (**B**) velocity (cm/s) during the open field test in mice. Drugs were administered 1 h, 3 h, and 5 h prior to the test. All drug-treated groups were compared with the respective vehicle-treated group (control). Each bar represents the mean ± SEM of 10 mice for each treatment. Statistical analysis was performed by two-way ANOVA followed by Tukey’s post hoc for multiple comparison among data set. * *p* < 0.05, ** *p* < 0.01, and *** *p* < 0.001, indicate a significant difference compared to controls, and + *p* < 0.05, and +++ *p* < 0.001 versus 1 h vehicle treated group.

Measurements after 1 h of drug administration (short protocol), revealed that distance traveled and velocity were significantly decreased under MDMB-CHMINACA and 5F-ADB-PINACA treatment (Figure 3A,B). After 3 h of drug administration, (long protocol) these effects persisted only in the MDMB-CHMINACA treated group, whereas low mobility effects were observed in the APICA- and 5F-ADB-PINACA-treated groups (Figure 3A,B). Five hours after drug treatment (extended protocol), APICA significantly decreased the distance travelled. Low mobility effects continued under 5F-ADB-PINACA and an apparent mobility increase was observed in the MDMB-CHMINACA treated group (Figure 3A,B).

#### 2.1.2. Anxiety-like Behavior

**Figure 4:**
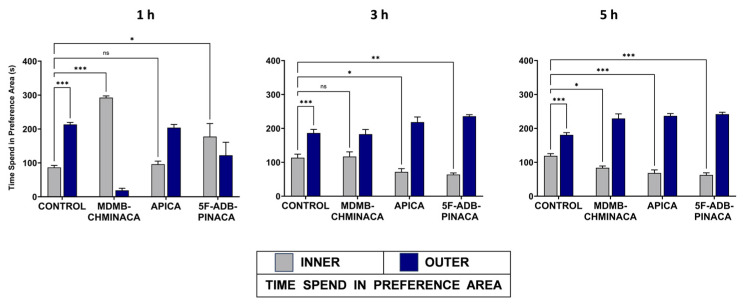
Effect of 1 mg/kg systemic administration of MDMB-CHMINACA, APICA, and 5F-ADB-PINACA on the anxiety-like behavior of mice. Anxiety-like behavior was measured as the amount of time (s) mice spent in the INNER area vs. the OUTER area of the arena during the OFT. Longer periods in the OUTER area reflect anxiety-like behavior. All drug-treated groups’ INNER fragments were compared to the INNER fragment of the vehicle-treated groups (control). Each bar represents the mean ± SEM of 10 mice for each treatment. Statistical analysis was performed by two-way ANOVA followed by Bonferroni’s post hoc for multiple comparison among data set * *p* < 0.05, ** *p* < 0.01, and *** *p* < 0.001, indicate significant differences compared to control. ns, not significant.

Anxiety-like behavior was examined as the difference in the period of staying at the center area of the arena (INNER) for the vehicle-treated group and those of the drug-treated groups. The control group exhibited anxiety-like behavior at all time points (longer periods of stay in the OUTER area) (Figure 4). The apparent overall preference for the OUTER area in the drug-treated groups, despite the effects of SC on mobility, can be presumed to be an anxiety-like behavior. The exception was MDMB-CHMINACA and 5F-ADB-PINACA at 1 h (Figure 4). One hour after drug administration, the MDMB-CHMINACA-treated group exhibited a longer period of staying in the INNER region (Figure 4), which responded to the immobile state owing to catalepsy. At 3 and 5 h, all drug-treated groups displayed significantly different preference rates for the INNER area compared with the control, except for MDMB-CHMINACA at 3 h and APICA at 1 h (Figure 4). This indicates that SCs can induce anxiety-like symptoms at different times after drug exposure.

#### 2.1.3. Memory and Learning

**Figure 5:**
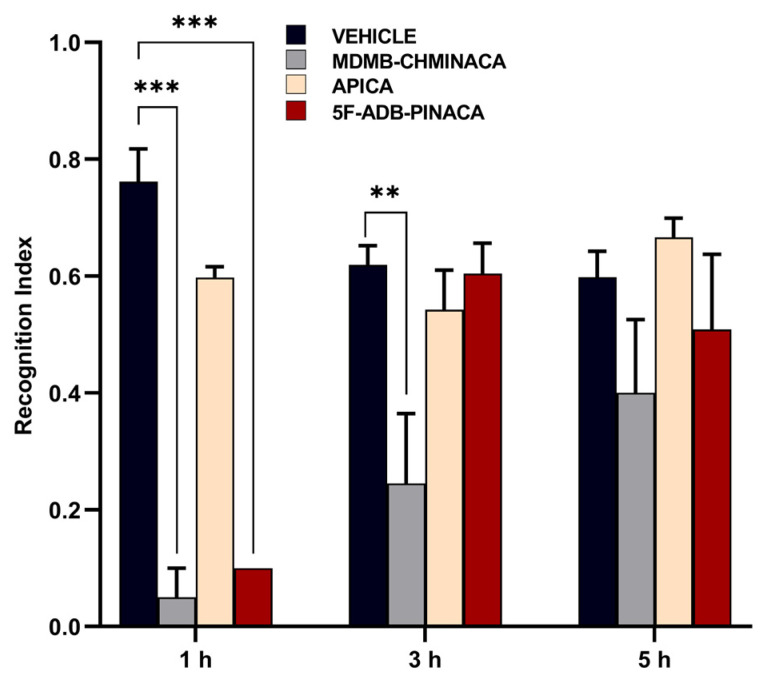
Effects of 1 mg/kg systemic administration of MDMB-CHMINACA, APICA, and 5F-ADB-PINACA on the recognition index for the novel object recognition test in mice. Trial is based on the index (%) of exploration and consequent preferences of mice toward a novel object compared to a previously familiarized object. Values closer to one indicate no memory and learning impairment and values below 0.5 suggest strong memory impairment. All drug treatments were administered intraperitoneally. Each bar represents the mean ± SEM of 10 mice for each treatment. Statistical analysis was performed by two-way ANOVA followed by Tukey’s post hoc for multiple comparison among data set. ** *p* < 0.01 and *** *p* < 0.001 indicate significant differences compared to control (vehicle).

To assess memory disruption, the effects of MDMB-CHMINACA, 5F-ADB-PINACA, and APICA on learning and memory consolidation were evaluated using the novel object recognition test (Figure 2B). MDMB-CHMINACA induced memory impairment in both the 1 h and 3 h protocols. In the case of 5F-ADB-PINACA, significant detrimental effects on learning were observed only in the 1 h protocol, with a tendency towards memory inhibition apparent at 5 h post-drug administration (Figure 5). APICA did not exert a significant impact on learning; nevertheless, a sustained decline in learning patterns was observed at 1 h and 3 h after drug administration (Figure 5).

### 2.2. Alteration of Brain Derived Neurotrophic Factor (BDNF) in the Hippocampus

**Figure 6:**
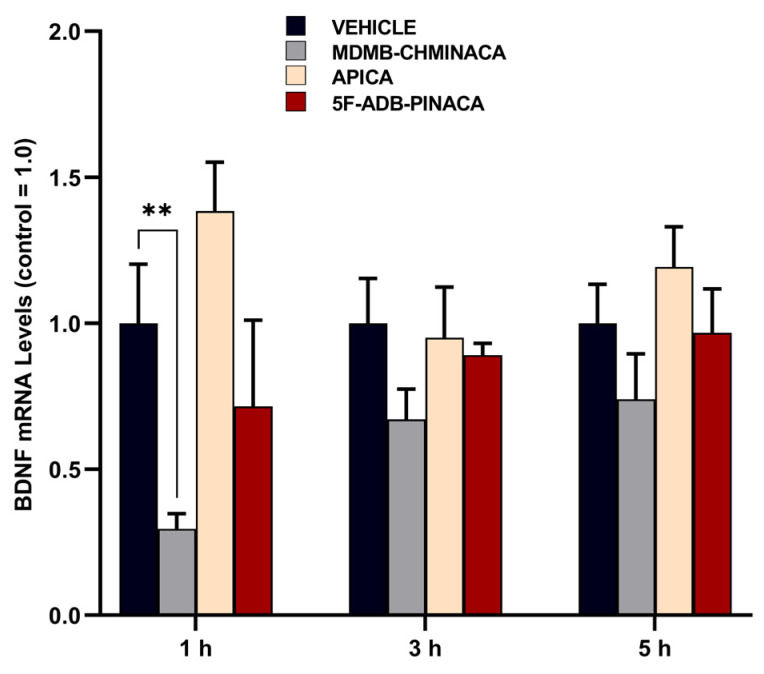
Effect of MDMB-CHMINACA, APICA, and 5F-ADB-PINACA on the mRNA expression levels of brain-derived neurotrophic factor (BDNF). Mice were administered 1 mg/kg of either MDMB-CHMINACA, APICA, 5F-DB-PINACA, or vehicle (control) at 1 h, 3 h, and 5 h, as shown. Their hippocampi were collected after treatment. The indicated/relative levels of mRNA were analyzed via quantitative reverse transcription real-time polymerase chain reaction (qRT-PCR) and normalized to those of β-actin. The bars represent the mean ± SEM of 5 mice. Statistical analysis was performed by two-way ANOVA followed by Tukey’s post hoc for multiple comparison among data set. ** *p* < 0.01 indicates significant difference compared to control.

The mRNA expression levels of brain-derived neurotrophic factor (BDNF) were significantly reduced by MDMB-CHMINACA at 1 h after treatment. This effect was not significant at the 3 h and 5 h time points (Figure 6).

### 2.3. Effects on Endocannabinoid Concentrations in the Hippocampus

**Figure 7:**
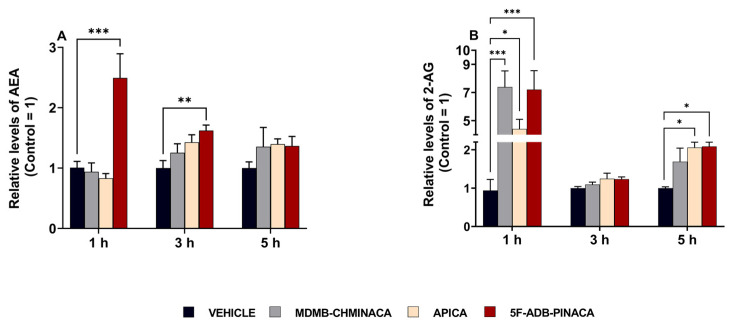
Quantification of the endogenous cannabinoids (**A**) AEA and (**B**) 2-AG content in hippocampal tissue of male C57BL/6J mice 1, 3, and 5 h after synthetic cannabinoid treatment. Each mouse was administered a 1 mg/kg dose of MDMB-CHMINACA, APICA, 5F-ADB-PINACA, or vehicle (control) as shown. Subsequently, endocannabinoid content was quantified via UPLC-TOF/MS. Each drug treatment was compared to its control (vehicle). Each bar represents the mean ± SEM of 5 mice for each treatment. Statistical analysis was performed by two-way ANOVA followed by Tukey’s post hoc for multiple comparison among data set. * *p* < 0.05, ** *p* < 0.01, and *** *p* < 0.001, indicate significant differences compared to control.

In the 1 h protocol, hippocampal AEA and 2-AG content after 5F-ADB-PINACA administration were elevated by approximately 2.5-fold and 7-fold, respectively, compared to the control (Figure 7A,B). Likewise, the 2-AG content in the hippocampus after MDMB-CHMINACA and APICA administration was increased 7-fold and 5-fold, respectively, compared to that of the control (Figure 7A,B). At 3 h after drug administration, a significant increase was only observed in AEA content after 5F-ADB-PINACA exposure (Figure 7A). There was an upward trend for both AEA and 2-AG in all of the synthetic cannabinoid treatments (Figure 7A,B). In the 5 h protocol, the upward trend in AEA content was still sustained among all SC-treated groups (Figure 7A). Moreover, a significant elevation in 2-AG content was observed in both the 5F-ADB-PINACA- and APICA-treated groups (Figure 7B).

### 2.4. Alteration of Endocannabinoids Metabolic Enzymes, FAAH and MAGL

**Figure 8:**
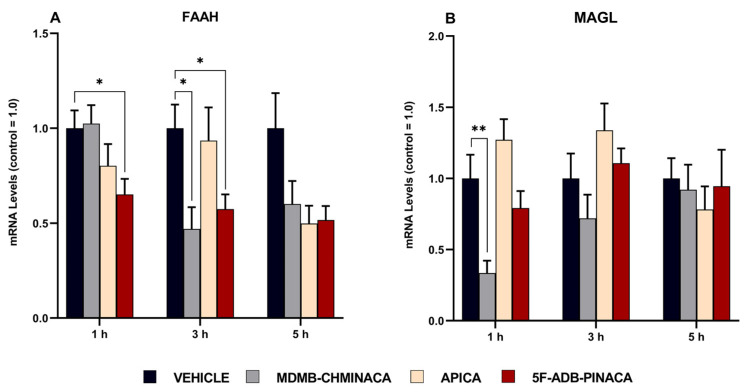
Effect of MDMB-CHMINACA, APICA, and 5F-ADB-PINACA on the mRNA expression of (**A**) AEA-degrading enzyme (FAAH) and (**B**) 2-AG degrading enzyme (MAGL). Mice were administered 1 mg/kg of MDMB-CHMINACA, APICA, 5F-DB-PINACA, or vehicle (control) at 1, 3, and 5 h, as shown. Their hippocampi were collected after treatment. The indicated/relative levels of mRNA were analyzed via quantitative reverse transcription real-time polymerase chain reaction (qRT-PCR) and normalized to those of β-actin. The bars represent the mean ± SEM of 5 mice. Statistical analysis was performed by two-way ANOVA followed by Tukey’s post hoc for multiple comparison among data set. * *p* < 0.05 and ** *p* < 0.01, indicate significant differences compared to control.

In the 1 and 3 h protocols, the mRNA expression levels of the AEA-degrading enzyme, fatty acid amide hydrolase (FAAH), were significantly reduced after 5F-ADB-PINACA exposure (Figure 8A); after MDMB-CHMINACA administration, a reduction was observed only at 3 h (Figure 8A). Similarly, MDMB-CHMINACA also reduced the mRNA expression levels of the 2-AG degrading enzyme, monoacylglycerol lipase (MAGL), in the 1 h protocol (Figure 8B). In the 5 h protocol, the mRNA expression levels of FAAH and MAGL were not significantly affected. However, a sharp reduction trend persisted among all drug-treated groups compared with that of the control group (Figure 8A,B).

## 3. Discussion

Among the three synthetic cannabinoids examined in this study, MDMB-CHMINACA, and 5F-ADB-PINACA significantly decreased the locomotive and velocity capabilities of mice at 1 h (Figure 3A,B). A cross-time comparison against the 1 h vehicle-treated group revealed that both MDMB-CHMINACA and 5F-ADB-PINACA sustained these effects after 3 h of drug administration, whereas APICA significantly decreased these parameters 5 h after drug administration (Figure 3A,B).

MDMB-CHMINACA and 5F-ADB-PINACA induced notable memory impairments at 1 h, with MDMB-CHMINACA causing sustained effects at 3 h (Figure 5). In contrast, APICA showed no significant memory impairment in all three study protocols (Figure 5). These results highlight the potential of novel SCs to impact affective, cognitive, and psychomotor functions [40]. The diverse chemical structures and substituents of SCs may influence the severity of these effects. Exemplified by APICA treatment, wherein significant motion impairment emerged 5 h post-exposure (Figure 3A,B), indicating that the progression of cognitive-related impairments could serve as a qualitative indicator of SC abuse.

Anxiety behavior was observed at 1 h for the APICA groups (Figure 4). The MDMD-CHMINACA-treated group developed strong catalepsy effects 1 h after drug exposure. Therefore, anxious behavior could not be adequately assessed. Strong catalepsy is a common and prevalent pharmacological effect of JWH-018 and MDMB-CHMICA derivatives [41]. Therefore, the observed preference for the INNER area of the arena 1 h after MDMB-CHMINACA administration alludes to catalepsy-induced immobility rather than anxiolytic effects (Figure 4). At 1 h, 5F-ADB-PINACA displayed a higher preference for the INNER area compared to the control group. Thus, anxiogenic effects can be assumed. However, 3 h and 5 h after drug administration, the APICA and 5F-ADP-PINACA groups exhibited significant anxiety-like behavior when compared to the control (Figure 4). Consequently, the findings indicate that novel SCs can induce anxiety-like symptoms in a long and extended timeframe of action. JHW-018 and its derivatives have been categorized as substances which lead to a high level of CB_1_ receptor activation, which results in anxiety, memory loss, and cognitive deficits [42,43]. Thus, both behavioral disruption and cognitive impairment may be suggested as a common pharmacological indicator of SC abuse. It is worth mentioning that anxiety effects could be induced as a response to high concentrations of AEA, as reported previously [44].

Cognitive processes, involving the learning and working memory, undergo encoding as the initial step in memory acquisition, followed by consolidation. Disruptions during acquisition can hinder encoding and impair consolidation [45,46]. BDNF, a protein crucial for higher cognitive processes such as working memory, plays a vital role in memory acquisition and learning [47,48]

In this study, mice exposed to MDMB-CHMINACA and 5F-ADB-PINACA showed reduced BDNF mRNA expression levels 1 h post-administration (Figure 6), impacting their learning memory and recognition (Figure 5). Since BDNF is an essential component in long-term memory processes [49], the observed marked reduction in BDNF, particularly under MDMB-CHMINACA and 5F-ADB-PINACA, at 3 and 5 h suggests potential negative effects on long-term memory consolidation (Figure 6). BDNF’s role in neuroplasticity modulation and adaptive processes [50,51] indicates that SCs, through alterations in BDNF, may disrupt early memory and learning acquisition, thus impairing memory consolidation.

The overall content of endocannabinoids AEA and 2-AG in the hippocampus was significantly increased 1 h after drug treatment (Figure 7A,B). The observed increase in 2-AG was higher than that of AEA, likely resulting from the higher innate levels of 2-AG with respect to those of AEA [52].

Three hours after drug administration, a significant increase in the EC content in the hippocampus was observed only for AEA after 5F-ADB-PINACA treatment (Figure 7A). Conversely, the content of 2-AG exhibited only a sustained increasing trend (Figure 7A). At 5 h after drug administration, the significant elevation in AEA content was no longer observed; however, elevated levels with regard to the control were still sustained and 2-AG content was significantly increased after APICA and 5F-ADB-PINACA administration (Figure 7B). AEA and 2-AG mediate their activities mostly through CB_1_ activation [15]. The SCs’ affinities towards the CB_1_ receptor adversely affect MAGL and FAAH, which in turn negatively affects locomotor functions, behavior, and synaptic plasticity [53,54]. Considering that SCs consisting of L-valinate and tert-leucinamide derivatives, such as MDMB-CHMINACA and 5F-ADB-PINACA (Figure 1), have reported lower EC50 (half maximum effective concentration) values for the CB_1_ receptor, indicating their high preference for the activation of this receptor [28,29,35], it is likely to suggest that the SCs’ strong affinities towards CB_1_ can disrupt EC content through MAGL and FAAH alterations.

The 2-AG content increased 5 h after APICA treatment (Figure 7B), aligning with previous studies characterizing APICA as less potent than JWH-018 [55], with a more prolonged duration of cannabimimetic effects and reduced immediate effects [28]. Metabolites of APICA have been reported to exhibit reduced activity at CB_1_, yet they are highly efficacious CB_1_ agonists [56]. Therefore, it is possible to assume that the locomotive impairment and endocannabinoid elevations in APICA treatment group at 5 h post-exposure may be attributed to these factors (Figure 3 and Figure 7B).

A significant decrease in the mRNA expression levels of FAAH was observed 1 h after 5F-ADB-PINACA administration. Three hours after drug exposure, a significant decrease was observed in both the MDMB-CHMINACA- and 5F-ADB-PINACA-treated groups (Figure 8A). In FAAH-knockout mice, an up to 10-fold increase in the AEA content in the brain has been reported [57]. This finding highlights the validity of our statement that synthetic cannabinoid exposure inhibits FAAH, resulting in an increase in AEA. In addition, when FAAH inhibitors are administered, both the activity and half-life of AEA have been reported to significantly increase in the long term [58]. As mentioned previously, elevated levels of AEA induce anxiety-like effects [44]. Consequently, it is reasonable to consider the downward trend in FAAH mRNA expression levels 5 h after drug administration (Figure 8A) as negatively influencing mobility and anxiety-like behaviors in the long term (Figure 3 and Figure 4). A study using MAGL-deficient mice reported a marked increase in 2-AG levels in the brain and peripheral tissues [53]. In our study, a decrease in MAGL mRNA expression levels was observed 1 h after MDMB-CHMINACA administration (Figure 8B). In the 3 h protocol, the MAGL mRNA expression levels were not significantly altered; still, a downward trend was observed (Figure 8B). For that reason, the increase in 2-AG content observed in the current study is thought to be closely related to MAGL suppression as an outcome of SC exposure. Moreover, elevated levels of 2-AG were associated with impaired decision making [59]. With that in mind, our study hypothesized that MDMB-CHMINACA exposure can lead to a high level of CB_1_ receptors activation and ECs alteration, particularly 2-AG, consequently leading to behavioral and cognitive disruption.

The SCs employed in this study exhibited diverse behavioral disruptions and distinct cognitive impairments, showcasing their erratic pharmacological nature. For instance, APICA demonstrated significant adverse effects at 5 h post-drug administration, while 5F-ADB-PINACA showed pronounced cognitive impairment only 1 h post-drug exposure. Our hypothesis associates these observed adverse effects with the structural modifications and metabolic instability of SCs, as well as the manifested time-dependent accumulation of ECs. Considering these results, our study emphasizes the importance of investigating SCs not solely in the short term but across different time periods following drug exposure.

To this end, the present study proposes that an increase in hippocampal EC content and a reduction in BDNF expression levels are intrinsic attributes associated with SC exposure, indicating a plausible relation between altered ECs and cognitive as well as behavioral disruptions. This implies the categorization of a state which is indicative of SC drug abuse.

In the context of the increasing use of SCs, where concerns related to cannabis recreational activities predominantly centers on legal consequences rather than perceived harm [60], our study provides scientific evidence elucidating the adverse biological implications of SC consumption, particularly in the alteration of ECs metabolism.

## 4. Materials and Methods

### 4.1. Materials

MDMB-CHMINACA (Cat. No. JWH 16200), APICA (Cat. No. CAY 9001193), and 5F-ADB-PINACA (Cat. No. CAY 14764) were purchased from Cayman Chemical (Ann Arbor, MI, USA) by the National Institute of Health Science, Japan, and were provided in response to a formal request submitted by the Division of Pharmaceutical Cell Biology, Graduate School of Pharmaceutical Sciences, Kyushu University, Fukuoka, Japan. Deuterated forms of AEA (5Z,8Z,11Z,14Z)-N-(2-hydroxy-[1,1,2,2-d4]ethyl)eicosa-5,8,11,14-tetraenamide and 2-AG (5Z,8Z,11Z,14Z)-eicosatetraenoic-5,6,8,9,11,12,14,15-d8 acid 2-glyceryl ester were purchased from Abcam (AEAd_4_: AB120446 and 2-AGd_8_: AB120919, Cambridge, UK). Acetonitrile LC/MS grade was purchased from Kanto chemicals Co, Inc. (Cat. No. 01033-76, Tokyo, Japan). Ethanol guaranteed reagent grade 95% (Cat No. 14711-15) was purchased from Nacalai Tesque, Inc., Kyoto, Japan. Tween-80 (polyoxyethylene sorbitan monooleate) (Cat No. T2533) was obtained from Tokyo Chemical Industry, Co., Ltd., Tokyo, Japan. All other reagents used were of the highest commercially available grade.

### 4.2. Animals and Treatment

Male C57BL/6J mice were obtained from CLEA Corporation (Tokyo, Japan) at 7 weeks of age and tested at 8 weeks of age. Mice were housed two and three per cage for 1 week after their arrival in a temperature- and humidity-controlled environment. The rearing room was controlled on a 12 h light–dark cycle (lights off at 7:00 p.m.), and the room temperature was maintained at 24 ± 2 °C. The mice were maintained on a commercial diet (CLEA Rodent Diet CE-2, CLEA, Shizuoka, Japan) with ad libitum access to food and water. The animal protocols were approved by the Institutional Animal Care and Experimental Committee of Kyushu University.

Drugs were administered intraperitoneally and injected (1.0 mg/kg) at a volume of 0.1 mL per 10 g of body weight (30 g). Dosage was stipulated based on our previous study involving a 1 mg/kg dose of synthetic cannabinoid JWH-018 [37] and in accordance with prior studies on behavioral effects of repeated administration of Δ^9^-THC, JWH-018, and its halogenated derivatives [61,62]. Drugs were initially prepared by individually dissolving the solid drug in 2% ethanol and 2% Tween-80 and brought to the final volume with saline (0.9% NaCl). The vehicle solution was composed of 2% ethanol, 2% Tween-80, and saline.

### 4.3. Overview of the Experimental Design

The effects of SCs MDMB-CHMINACA, 5F-ADB-PINACA, and APICA on behavior (locomotive and anxiety) and cognition (recognition index) were assessed based on the open field and novel object recognition (NOR) tests, with some modifications [37,63,64]. Two separate and independent experiments were conducted: one for exploration and anxiety-like behavior and another for cognitive impairment. Subjects were tested after 1, 3, or 5 h of drug or vehicle administration (short-, long-, and extended-time-frame protocols, respectively). Daily evaluations were performed for each drug or vehicle. A new group of test subjects was employed for each treatment (*n* = 10) while maintaining consistent experimental conditions.

To minimize stress and anxiety, mice were handled daily for one minute during the week preceding the experiment. On the day of the experiment, the mice were acclimated to a new room for one hour [65]. To prevent olfactory cues, objects and apparatus were thoroughly cleaned with an ethanol (70%) solution between animal trials and during different phases of the novel object recognition test [64,66,67]. Male mice were used to avoid the estrous cycle fluctuations in females [68]. The sample size was determined based on previous studies [37,61] and guidelines for behavioral experiments with laboratory animals [67,68,69]. All procedures adhered to the National Institutes of Health (NIH) Guidelines for Animal Care and Ethical Conduct.

After completing behavioral and recognition trials for each designated timeframe, tissue sampling was performed. Hippocampus was selected for its integral role in memory processing, recognition, and acquisition functions [70,71], and high density of CB_1_ receptors [72] that bind to both ECs and SCs. Mice were euthanized for hippocampal collection, and tissues were immediately placed in liquid nitrogen and frozen at −80 °C.

#### Behavioral Response Assessment

The open field was selected as it is a non-stressful and useful test focused on novel environment exploration that does not rely on disruptors, such as objects, social interaction, starvation and water restriction regimens, or punishment techniques facilitating the observation of natural anxiety-like responses in mice rather than that induced by fear or despair [63,73,74,75].

Novel object test was chosen because it facilitates the study of both rodents’ curiosity and innate spontaneous behavior towards novel objects, enhancing working memory [47,64,67]. Diverse intervals between phases serve as indicators of the specific type of memory under examination. Additionally, distinct timings of pharmacological interventions promote the exploration of potential factors that influence the processes of memory formation and modulation [76].

##### Exploration

Short-term protocol assessments were performed 1 h after drug or vehicle administration. Long and extended protocol assessments were performed 3 and 5 h after drug or vehicle administration, respectively. Each mouse was allowed to freely explore an open-field box (60 cm × 60 cm) with black vertical walls and a white floor. Their behavior was recorded for analysis as the total distance traveled (cm) and velocity (cm/s) in 5 min (Figure 2A).

##### Anxiety

Anxiety behavior was monitored as the average amount of time test subjects spent in the center area (INNER) versus the walls or border regions of the arena (OUTER) during the open field test (Figure 2A).

##### Recognition Index

Test subjects were evaluated for novel object recognition at 1, 3, or 5 h after drug or vehicle administration. After treatment, a 5 min arena exploration was allowed at each time point. Subsequently, a 5 min familiarization trial with two familiar objects (FAMILIAR X and X) was executed, followed by a 5 min test trial in which familiar object X was replaced with novel object Y. The intervals between familiarization, and test trials were 45 min, 1 h, and 2 h, (short-, long-, and extended-time-frame protocols, respectively). The recognition index (RI) was recorded as the proportion of time spent exploring the novel Y to the cumulative time spent exploring Y and X. (RI) = (novel Y)/(novel Y + familiar X) (Figure 2B).

### 4.4. Determination of Endocannabinoid AEA & 2-AG Contents in Hippocampus

Analysis was performed using UPLC-TOF-MS manufactured by Waters™, with the following configuration: Waters LCT Premier™ mass spectrometer (Waters Corp., Manchester, UK) (positive mode) equipped with an ACQUITY UPLC system (Waters Corporation, Milford, MA, USA), electrospray ionization (ESI+) detector, and an ACQUITY UPLC HSS T3-C18 column.

Vehicle- and drug-treated group samples were analyzed according to their respective timeframe protocols. The samples were prepared and quantified according to previously established methods [77]. Frozen hippocampal tissue (30 mg) was transferred to a borosilicate tube. and was homogenized with 1 mL of ice-cold acetonitrile spiked with 10 µL of an internal standard solution of 2 pmol AEA-d4 or 5 nmol 2-AG-d8. The homogenized hippocampus with internal standard was sonicated in ice-cold water for 30 min. The homogenates were kept at −20 °C overnight for protein precipitation. The samples were then centrifuged at 916× *g* (3000 rpm) (Kitman-18; TOMY KOGYO Co., Ltd., Tokyo, Japan) for 3 min. The supernatant was dried under a stream of N2, reconstituted with 300 µL of methanol/acetonitrile (2:1 *v*/*v*), and evaporated. The obtained sample was re-dissolved in 100 µL of acetonitrile, filtered, and subjected to ultra-performance liquid chromatography-time-of-flight mass spectrometry analysis. The measurement conditions for LC were as follows: apparatus: UPLC-TOF/MS(LCT-Primer XE; Waters, Milford, CT, MA); column: ACQUITY UPLC HSS T3 C18 column (2.1 × 100 mm, 1.7 mm i.d.; Waters); column temperature: 40 °C; sample temperature: 4 °C. Mobile phase: water, 10 mM ammonium acetate, 0.1% formic acid (solvent A), and acetonitrile (solvent B). Gradient: [% of B in A (min)] 2% (0–3), 2–100% (3–20), 100% (20–22), 100–2% (22–23), 2% (23–25). The flow rate was at 0.3 mL/min.

The MS analysis conditions were set as follows: source temperature, 120 °C; cone gas flow, 50 L/h; desolvation gas temperature, 350 °C; desolvation gas flow, 650 L/h; and capillary voltage, 3000 V in positive ESI mode. A lock mass of leucine–enkephalin was employed via a lock–spray interface to ensure accuracy during quantitative analysis.

### 4.5. Real Time-Reverse Transcription (RT)-Polymerase Chain Reaction (PCR)

Total RNA was extracted from the frozen hippocampus using RNeasy kits (Qiagen, Hilden, Germany), according to the manufacturer’s instructions. The total RNA concentration after extraction was quantified using Nano Vue (GE Healthcare, Chicago, IL, USA). The extracted total RNA was stored at −80 °C until use. The extracted total RNA was subjected to genomic DNA removal and reverse transcription using the PrimeScript^®^ RT reagent Kit with gDNA Eraser (Perfect Real-Time) according to the manufacturer’s instructions. Primer design and PCR conditions have been described previously [37]. Relative mRNA expression was determined using the Delta–Delta Ct method. The amount of quantified target mRNA was normalized to β-actin mRNA and is shown as a ratio relative to the control. (The control group was normalized to β-actin to a value of 1).

### 4.6. Data Analysis

For behavioral (exploration) studies, endocannabinoid quantification, and measurement of mRNA expression levels, two-way analysis of variance (ANOVA) was used to determine significant differences among the means of the treated groups (Control/MDMB-CHMINACA/APICA/5F-ADB-PINACA) followed by Tukey’s post hoc test. To measure anxiety-like effects, a two-way analysis of variance was performed followed by Bonferroni’s post hoc test to determine significant differences between the fractions in the inner area for the drug-treated groups and the vehicle-treated group.

For all comparisons, * *p* < 0.05, ** *p* < 0.01, and *** *p* < 0.001 were considered to indicate statistical significance. Statistical analyses were performed using GraphPad Prism 8 (GraphPad Software, San Diego, CA, USA).

## Data Availability

Data are available on request from the authors of individual results or corresponding authors.

## Abbreviations

MDMB-CHMINACA: N-[[1-(cyclohexylmethyl)-1H-indazol-3-yl]carbonyl]-3-methyl-L-valine, methyl ester; 5F-ADB-PINACA: N-(1-amino-3,3-dimethyl-1-oxobutan-2-yl)-1-(5-fluoropentyl)-1H-indazole-3-carboxamide; APICA: N-(1-adamantyl)-1-pentyl-1H-indole-3-carboxamide; EC: endocannabinoids; SC: synthetic cannabinoid; AEA: anandamide; 2-AG: 2-arachidonoyl glycerol; FAAH: fatty acid amide hydrolase; MAGL: monoacylglycerol lipase; BDNF: brain-derived neurotrophic factor; CB_1_R: cannabinoid receptor type 1; EMCDDA: European Monitoring Centre for Drugs and Drug Addiction.
